# Effect of Triple Combination Therapy With Lopinavir-Ritonavir, Azithromycin, and Hydroxychloroquine on QT Interval and Arrhythmic Risk in Hospitalized COVID-19 Patients

**DOI:** 10.3389/fphar.2020.582348

**Published:** 2020-10-08

**Authors:** Vincenzo Russo, Andreina Carbone, Filiberto Fausto Mottola, Rosa Mocerino, Raffaele Verde, Emilio Attena, Nicoletta Verde, Pierpaolo Di Micco, Luigi Nunziata, Francesco Santelli, Gerardo Nigro, Sergio Severino

**Affiliations:** ^1^Chair of Cardiology, Department of Translational Medical Sciences, University of Campania "Luigi Vanvitelli"—Monaldi Hospital, Naples, Italy; ^2^Cardiology Unit, Cotugno Hospital, Naples, Italy; ^3^Italy Medicine Unit, Division of Cardiology, San Giuliano Hospital, Naples, Naples, Italy; ^4^Department of Cardiology, Fatebenefratelli Hospital of Naples, Naples, Italy; ^5^Cardiology Unit, Boscotrecase Hospital, Naples, Italy; ^6^Department of Political Sciences, University of Naples Federico II, Naples, Italy

**Keywords:** coronavirus disease 2019, arrhythmias, QTc prolongation, SARS-CoV2, lopinavir/ritonavir, torsades des pointes

## Abstract

**Introduction:**

No data are provided about the effect of triple combination therapy with Lopinavir/Ritonavir (LPN/RTN), hydroxychloroquine (HQ) and azithromycin (AZT) on corrected QT (QTc) interval and arrhythmic risk, in COVID-19 patients. This study aims to describe the incidence of extreme QTc interval prolongation among COVID-19 patients on this experimental treatment and to identify the clinical features associated with extreme QTc prolongation.

**Materials and Methods:**

Data of 87 COVID-19 patients, treated with triple combination including LPN/RTN, HQ and AZT, were analyzed. QT interval was obtained by the tangent method and corrected for heart rate using Bazett’s formula. Extreme QTc interval prolongation was considered an absolute QTc interval ≥ 500 ms or an increase in QTc intervals of 60 ms or greater (ΔQTc ≥ 60 ms) compared with baseline.

**Results:**

Hypertension (66.7%) and diabetes (25.3%) were the most prevalent cardiovascular comorbidities. Twenty patients (23%) showed extreme QTc interval prolongation; no clinical, electrocardiographic or pharmacological characteristics have been associated to extreme QTc prolongation, except the history of ischemic stroke (*P= 0,007*). One torsade de pointes (TdP) in patient with QTc extreme prolongation (QTc: 560 ms) after 5 days of therapy was recorded.

**Conclusions:**

We observed a high incidence of extreme QTc interval prolongation among COVID-19 patients on triple combination therapy. Since the incidence of malignant arrhythmias seems to be not negligible, a careful electrocardiographic monitoring would be advisable.

## Highlights

1. COVID -19 treatment was associated to electrocardiographic alterations.
2. High incidence of extreme QTc interval prolongation among COVID-19 patients was associated to triple combination therapy with lopinavir-ritonavir, azithromycin and hydroxychloroquine.
3. Since the incidence of life-tethering arrhythmias seems to be not negligible, a careful electrocardiographic monitoring would be advisable in COVID-19 patients.

## Introduction

Coronavirus disease 2019 (COVID-19) outbreak is a whole earth health emergency related to a highly pathogenic human coronavirus responsible of severe acute respiratory syndrome (SARS-CoV-2). The pharmacological treatments currently administered in patients with COVID‐19 include antiviral agents, as Lopinavir/Ritonavir (LPN/RTN), and drugs with immune‐modulatory and/or anti‐inflammatory properties, as hydroxychloroquine (HQ) and azithromycin (AZT), which are currently off-label used in Chinese and Italian centers (Dong et al., 2020; Russo et al., 2020a). LPN/RTN causes the dose-dependent block of both human ether-a-go-go-related gene (HERG) potassium channels and delayed rectifier potassium current (IKr) channels predisposing to QT prolongation and torsade de pointes (TdP) (Anson et al., 2005; Russo et al., 2020b). Increased corrected QTc interval (QTc) has been reported in hospitalized COVID-19 patients treated with HQ/AZT (Bun et al., 2020; Chorin et al., 2020; Cipriani et al., 2020; Hor et al., 2020; Mercuro et al., 2020; Ramireddy et al., 2020) alone or in combination, due the inhibitory effects on IKr channels. The concomitant use of three or more QT prolonging drugs twice increases the risk of QT prolongation in hospitalized patients on cardiology ward (Khan et al., 2017); however, no data on the effect of triple combination therapy with LPN/RTN, HQ and AZT on corrected QT (QTc) interval and arrhythmic risk have been still provided. The aim of our study was to describe the incidence of extreme QTc interval prolongation among COVID-19 patients on this experimental treatment; and to identify the clinical features associated with extreme QTc prolongation.

## Materials and Methods

This is an observational, retrospective, multicenter study including consecutive laboratory-confirmed COVID-19 patients admitted at 3 hospitals in Italy between March 1 and April 1, 2020, who were treated with triple combination including LPN/RTN (250/50 mg twice daily), HQ (200 mg twice daily) and AZT (500 mg once daily) and who underwent electrocardiographic evaluation before and during the pharmacological therapy, according to the hospital's clinical practice. The laboratory confirmation was achieved by real time quantitative reverse-transcription polymerase chain reaction (RT-PCR) assay on nose/throat swab or sputum sample positive for SARS-CoV-2. The electrocardiographic examinations performed before and during the pharmacological therapy according to the hospital's clinical practice have been collected. Electrocardiographic measurements were manually performed by expert physicians; QT interval was obtained by the tangent method and corrected for heart rate using Bazett’s formula (QTc=QT/√RR) (Postema et al., 2008). The incidence of extreme QTc interval prolongation and arrhythmias during the pharmacological treatment has been reported. Extreme QTc interval prolongation was considered an absolute QTc interval ≥ 500 ms or an increase in QTc intervals of 60 ms or greater (ΔQTc ≥ 60 ms) compared with baseline (Drew et al., 2010). The institutional ethics committee approved the study.

### Statistical Analysis

Distribution of continuous data was tested with the Kolmogorov–Smirnov and the Shapiro-Wilk test. Normally distributed variables were expressed as mean ± standard deviation (SD), whereas non-normal distributed ones as median and interquartile range (IQR). Categorical variables were reported as numbers and percentages. The unadjusted (univariable) and adjusted (multivariable) risk ratios (RR) both for incident extreme QTc prolongation were calculated using logistic regression models and presented as RR with their 95% confidence intervals (CI). All independent variables showing a p value <0.1 for the association with the response variable at univariable analysis were tested in the multivariable model. For all tests, a p value <0.05 was considered statistically significant. Analyses were performed by using R version 3.5.1 (R Foundation for Statistical Computing, Vienna, Austria).

## Results

Eighty-seven hospitalized COVID-19 patients (median age 65± 14, 61 % male) treated with triple combination including LPN/RTN, HQ, and AZT were included in the present analysis. Hypertension (66.7%) and diabetes (25.3%) were the most prevalent cardiovascular comorbidities. 16 patients (18.4%) were mechanically ventilated. The baseline characteristics of study population are shown in **Table 1**.

**Table 1:** Baseline characteristic of overall study population.

Variable	
N	87
Age (mean ± SD)	65±14
Gender. male (%)	53 (60.9)
BMI> 30 kg/m^2^, n (%)	15 (17.2)
HR (bpm) median (IQR)	75 (47–150)
QRS (ms) median (IQR)	90 (80-120)
I degree AV block, n (%)	7 (7.7)
Smokers, n (%)	17 (19.5)
AF, n (%)	11 (12.6)
Dyslipidemia, n (%)	25 (28.7)
Hypertension, n (%)	58 (66.7)
Diabetes Mellitus, n (%)	22 (25.3)
CAD, n (%)	11 (12.6)
DCM, n (%)	4 (4.6)
Previous ischemic stroke, n (%)	8 (9.2)
CKD, n (%)	9 (10.3)
COPD, n (%)	13 (14.9)
Orotracheal intubation, n (%)	16 (18.4)
**Medical therapy:**	
- ACEI/ARBs	34 (39)
- Ca-antagonists, n (%)	17 (19.5)
- Beta-blockers, n (%)	24 (27.6)
- Alfa-blockers, n (%)	4 (4.6)
- Amiodarone, n (%)	3 (3.4)
- IC class AA drugs, n (%)	3 (3.4)
- Antiplatelet drugs, n (%)	21 (24.1)
- Statin, n (%)	20 (23)
- Anticoagulant, n (%)	8 (9.2)
- Digoxin, n (%)	3 (3.4)

BMI, body mass index; HR, heart rate; AV, atrio-ventricular; AF, atrial fibrillation; CAD, coronary artery disease; CKD, chronic kidney disease; COPD, chronic obstructive pulmonary disease; DCM, dilated cardiomyopathy; ACEI, angiotensin converting enzyme inhibitor; ARBs, angiotensin II receptor blockers; AA, antiarrhythmic.

**Figure 1:**
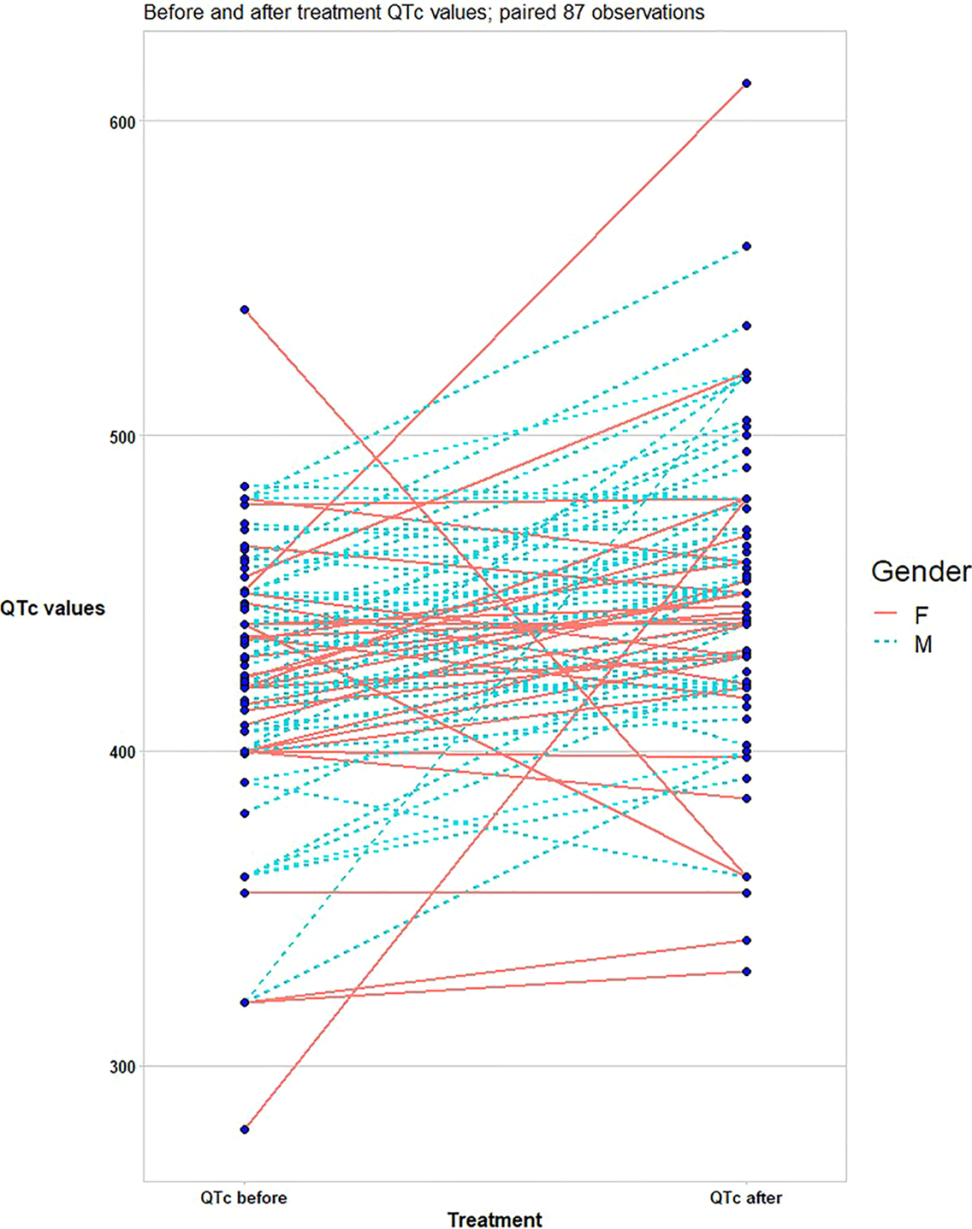
Difference in corrected QT (QTc) between COVID-19 patients at baseline and after triple combination therapy (lopinavir-ritonavir, azithromycin, and hydroxychloroquine).

The QTc interval prolonged from baseline median value (IQR) of 422 (280–40) ms (ms) to a maximum median (IQR) value of 450 (330–612) ms after 6.5 (IQR 4–9) days of therapy. 20 patients (23%) showed extreme QTc interval prolongation; in particular, 12 patients (13.8%) showed an absolute QTc interval ≥ 500 ms; 18 patients (20.7%) and ΔQTc≥ 60 ms and 10 patients (11.5%) both QTc and ΔQTc prolongation (**Figure 1**). No clinical, electrocardiographic or pharmacological characteristics have been associated to extreme QTc prolongation, except the history of ischemic stroke (*P= 0,007*) (**Table 2**).

**Table 2:** Distribution of patients’ characteristics and their association with extreme corrected QT (QTc) prolongation among COVID-19 study population.

Variable	Extreme QTc prolongation	No QTc prolongation	Univariate	Multivariate
			RR (CI), p value	RR (CI), p value
N	20	67		
Age (mean ± SD)	66±10.2	64 ± 13.3	1 (0.9–1.04); p:0.85	–
Gender. male (%)	16 (80)	37 (55)	3.2 (0.9–10); p:0.05	3.9 (0.9–16); p:0.06
BMI> 30 kg/m^2^ . n (%)	3 (15)	12 (17.9)	0.8 (0.2–3); p:0.80	–
HR (bpm) median (IQR)	76 (47–150)	82 (50–137)	1.01(0.9–1.04); p: 0.18	–
QRS (ms) median (IQR)	90 (80–100)	90 (80–110)	1.01 (0.96–1.07); p:0.54	–
I degree AV block. n (%)	2 (11.8)	3 (4.3)	2.7 (0.5–13); p: 0.20	–
Smokers. n (%)	1 (5)	16 (23.5)	0.16 (0.021–1.3); p:0.09	0.089 (0.007–1.18); p: 0.07
AF. n (%)	3 (15)	8 (11.9)	1.3 (0.3–5.4); p:0.71	–
Dyslipidemia. n (%)	7 (35)	18 (26.9)	1.46 (0.50–4.2); p: 0.48	–
Hypertension. n (%)	13 (65)	45 (67)	0.9 (0.3–2.6); p:0.85	–
Diabetes Mellitus. n (%)	6 (30)	16 (23.9)	1.37 (0.4–4.1); p:0.58	–
CAD. n (%)	5 (25)	6 (9.1)	3.4 (0.9–12); p: 0.07	2.3 (0.5–10); p:0.26
DCM. n (%)	2 (10)	2 (3)	3.6 (0.4–27); p: 0.21	–
Previous ischemic stroke. n (%)	5 (25)	3 (4.5)	7 (1.5–33); p: 0.012	14 (2–101); p: 0.007
CKD (dialysis). n (%)	3 (25)	6 (9)	1.8 (0.40–8); p: 0.44	–
COPD. n (%)	2 (10)	11 (16.4)	0.6 (0.1–2.7); p:0.50	–
Orotracheal intubation. n (%)	6 (30)	10 (14.9)	2.4 (0.7–7.8); p: 0.13	–
**Medical therapy:**				
- ACEs/ARBs. n (%)	6 (30)	28 (32)	0.9 (0.3–3); p:0.97	–
- Ca-antagonists. n (%)	5 (5)	12 (17.9)	0.27 (0.032–3); p: 0.22	–
- Beta-blockers. n (%)	5 (25)	19 (28.4)	0.24 (0.01–3); p: 0.40	–
- Alfa-blockers. n (%)	1 (5)	3 (4.5)	1.1 (0.1–11); p: 0.92	–
- Amiodarone. n (%)	0	3 (4.5)	p:0.99	–
- IC AA drugs. n (%)	0	3 (4.5)	p:0.99	–
- Antiplatelet drugs. n (%)	7 (35)	14 (20.9)	2 (0.7–6); p:0.2	–
- Statin. n (%)	4 (20)	16 (23.9)	0.8 (0.2–2.7); p:0.7	–
- Anticoagulant. n (%)	2 (10)	6 (9)	1.1 (0.2–6); p: 0.88	–
- Digoxin. n (%)	1 (5)	2 (3)	1.7 (0.14–19); p: 0.66	–

BMI, body mass index; HR, heart rate; AV, atrio-ventricular; AF, atrial fibrillation; CAD, coronary artery disease; CKD, chronic kidney disease; COPD, chronic obstructive pulmonary disease; DCM, dilated cardiomyopathy; ACEI, angiotensin converting enzyme inhibitor; ARBs, angiotensin II receptor blockers; AA, antiarrhythmic.

Among study population, two significative arrhythmic events were recorder: one torsade de pointes (TdP) in patient with QTc extreme prolongation (QTc: 560 ms) after 5 days of therapy; one complete AV block in patient with baseline first degree AV block (PR: 230 ms) after 3 days of therapy. Among patients with extreme QTc prolongation, AZT has been suspended in six patients (7%), whereas HY was stopped in four patients (4.5%) after a median 7 days (IQR 4–8).

A total of seven patients died during the hospitalization. The crude incidence rate of in-hospital mortality was 15% (n: 3) in QTc extreme prolongation group vs 5.9% (n: 4) in patients without *(P= 0.97)*. Not survived COVID-19 patients were older (*P= 0.016)* and more frequent male *(P= 0.04)* than those who survived and showed higher prevalence of coronary artery disease *(P= 0.002)*, CKD *(P= 0.011)*, dilated cardiomyopathy *(P= 0.001*) and previous stroke/TIA *(P= 0.007)* (**Table 3**). No statistically significant differences in incident extreme QTc prolongation, AZT or HCQ suspension have been found between the two groups.

**Table 3:** Distribution of patients’ characteristics and their association with death occurrence among COVID-19 study population.

Variable	Survived	Not-Survived 4	Univariate	Multivariate
			RR (CI), p value	RR (CI), p value
n	80 (92)	7 (8)		
Age (mean ± SD)	64±14	74±9	1 (0.9–1.1); p: 0.016	
Gender. male (%)	47 (59)	6 (85.7)	4 (0.5–36); p: 0.193	–
BMI> 30 kg/m^2.^n (%)	15 (19)	0	P: 0.99	–
HR (bpm) median (IQR)	75 (47–150)	86 (72–110)	1.02 (0.9–1.06); p:0.15	–
QRS (ms) median (IQR)	90 (80–120)	110 (90–120)	1.1 (1.03–2); p: 0.003	6 (0.11–355); p:0.37
I degree AV block. n (%)	6 (75)	1 (14)	2 (0.2–19); p: 0.53	–
Smokers. n (%)	16 (20)	1 (14.3)	0.7 (0.007–6); p: 0.71	–
AF. n (%)	9 (11.3)	2 (28.6)	3 (0.5–18); p: 0.20	–
Dyslipidemia. n (%)	23 (29)	2 (28.6)	0.9 (0.17–5.4); p:0.9	–
Hypertension. n (%)	53 (66)	5 (71.4)	1.2 (0.2–7); p:0.78	–
Diabetes Mellitus. n (%)	19 (24)	3 (43)	2.4 (0.5–11); p:0.27	–
CAD. n (%)	7 (9)	4 (57)	13 (2.5–7.5); p: 0.002	2.28 (0.03–163); p:0.70
DCM. n (%)	1 (1.3)	3 (43)	59 (4–704); p:0.001	3.2 (0.001–143); p:0.78
Previous ischemic stroke. n (%)	5 (6.3)	3 (43)	11 (1.9–64); p: 0.007	10 (0.3–383); p: 0.19
CKD (dialysis). n (%)	6 (7.5)	3 (25)	9 (1.7–51); p: 0.011	2.8 (0.09–87); p: 0.55
COPD. n (%)	13 (16.3)	0	P: 0.99	–
Orotracheal intubation. n (%)	11 (14)	5 (71.4)	15 (2–91); p: 0.002	16 (0.7–373); p: 0.076
Extreme QT prolongation. n (%)	17 (22)	3 (43)	2.7 (0.56–13); p:0.20	–
QTc≥500. n (%)	11 (14)	1 (14.3)	1 (0.1–9); p: 0.9	–
Delta QTc ≥ 60 ms. n (%)	15 (19)	3 (43)	3 (0.6–16); p:0.11	–
AZT suspension. n (%)	6 (7.5)	0	P: 0.99	–
HQ suspension. n (%)	3 (4)	1 (14.3)	4 (0.38–47); p:0.23	–
**Medical therapy:**				
- ACEs/ARBs. n (%)	30 (37.5)	3 (43)	4.5 (0.9–22); p:0.06	0.5 (0.008–30); p: 0.74
- Ca-antagonists. n (%)	15 (19)	2 (29)	1.7 (0.3–9); p:0.53	–
- Beta-blockers. n (%)	21 (26)	3 (43)	2.1 (0.4–10); p: 0.35	–
- Alfa-blockers. n (%)	4 (5)	0	P:0.99	–
- Amiodarone. n (%)	3 (4)	0	P:0.99	–
- IC AA drugs. n (%)	1 (1.3)	2 (29)	31 (2.4–410); p: 0.008	8 (0.064–1070); p:0.4
- Antiplatelet drugs. n (%)	21 (26.3)	0	P: 0.99	–
- Statin. n (%)	18 (22.5)	2 (29)	1.3 (0.2–7); p:0.7	–
- Anticoagulant. n (%)	5 (6.3)	3 (43)	11 (1.9–64); p: 0.007	3 (0.42–228); p: 0.60
- Digoxin. n (%)	1 (1.3)	2 (29)	31 (2.4–410); p: 0.008	1.5 (0.01–2091); p:0.90

BMI, body mass index; HR, heart rate; AV, atrio-ventricular; AF, atrial fibrillation; CAD, coronary artery disease; CKD, chronic kidney disease; COPD, chronic obstructive pulmonary disease; DCM, dilated cardiomyopathy; AZT, azithromycin; HQ, hydroxychloroquine; ACEI, angiotensin converting enzyme inhibitor; ARBs, angiotensin II receptor blockers; AA, antiarrhythmic.

## Discussion

The main findings of this study can be summarized as follows: the triple combination therapy with LPN/RTN, HQ, and AZT led to extreme QTc interval prolongation in 23% of hospitalized COVID-19 patients; the extreme QTc prolongation was responsible for a premature discontinuation of treatment in 11.5 % of patients; the incidence of TdP was 1.14% among study population; both the extreme QTc prolongation and premature discontinuation of therapy did not impact on in-hospital mortality.

Previous retrospective studies including different cohorts of COVID-19 patients showed that both AZT and cloroquine (CQ) or HQ increased the risk of QTc prolongation, with a incidence ranging from 10.7% to 36 % (Bessiere et al., 2020; Mercuro et al., 2020; Ramireddy et al., 2020).

The association of CQ/HQ and AZT resulted in a significantly greater increase in the QTc interval when compared with monotherapy with either CQ or HQ (Mercuro et al., 2020; Ramireddy et al., 2020; Saleh et al., 2020); however, though patients experienced QTc interval prolongation, especially when combination therapy was used, the risk of arrhythmic death and TdP were not increased (Bun et al., 2020; Mercuro et al., 2020).

LPN/RTN has been proposed as experimental therapy for improving the clinical outcome of hospitalized patients with mild/moderate COVID-19 over supportive care (Li et al., 2020). Its use in other clinical setting has been associated with PR interval prolongation, advanced-degree atrioventricular (AV) block, QTc interval prolongation, and TdP.

Actually, no data are still available about the incidence of QTc interval prolongation and life-tethering arrhythmias among COVID-19 patients on triple combination therapy with LPN/RTN, CQ/HQ and AZT. According our results, this association does not increase the risk of extreme QTc prolongation compared than reported with the association of AZT and CQ/HQ. The incidence of TdP among different cohort of COVID-19 patients on pharmacological treatment with AZ and CQ/HQ ranged from 0.4% (Bun et al., 2020; Chorin et al., 2020) to 1.9% (Mercuro et al., 2020); our findings confirmed the not negligible risk of TdP among COVID-19 patients on triple combination therapy with LPN/RTN, HQ and AZT. We found an independent association between previous ischemic stroke and extreme QTc prolongation. This data might be explained by the possible link between brain damage and QTc prolongation (Oppenheimer et al., 1992; Oppenheimer, 2006; Marafioti et al., 2014), as suggested by previous experimental animal models showing a possible implication of insula cortex ischemic damage in the autonomic modulation of ventricular repolarization (Wang et al., 2009).

The extreme QTc prolongation led to premature discontinuation of triple combination therapy in 11.5 % of patients, slightly increased than those reported (2.8%–11%) from previous studies using monotherapy or combination therapy with either CQ or HQ (Bun et al., 2020; Mercuro et al., 2020). Finally, among our study population, neither the extreme QTc prolongation, nor premature discontinuation of AZ or HQ were associated to in-hospital mortality. Our results should be interpreted in light of the limitations related to the retrospective observational nature of the study; larger multicenter prospective studies are required to confirm our preliminary findings.

## Conclusions

We observed a high incidence of extreme QTc interval prolongation among COVID-19 patients on triple combination therapy with lopinavir-ritonavir, azithromycin and hydroxychloroquine, leading to discontinuation of pharmacological treatments in about half the cases. Since the incidence of life-tethering arrhythmias seems to be not negligible, a careful electrocardiographic monitoring would be advisable in COVID-19 patients to early detect PR and QTc interval changing during pharmacological treatments.

## Data Availability Statement

The raw data supporting the conclusions of this article will be made available by the authors, without undue reservation, to any qualified researcher.

## Ethics Statement

Ethical review and approval was not required for the study on human participants in accordance with the local legislation and institutional requirements. Written informed consent for participation was not required for this study in accordance with the national legislation and the institutional requirements.

## Author Contributions

VR and AC conceived the paper. FM, NV, RM, and RV collected the data. EA, PM, and FS analyzed the data. GN and SS performed the revision. All authors contributed to the article and approved the submitted version.

## Conflict of Interest

The authors declare that the research was conducted in the absence of any commercial or financial relationships that could be construed as a potential conflict of interest.
